# Enhanced Recovery After Surgery (ERAS) Protocol in Geriatric Hip Fractures: An Observational Study

**DOI:** 10.7759/cureus.42073

**Published:** 2023-07-18

**Authors:** Mohamed Sameer, Sathish Muthu, PC Vijayakumar

**Affiliations:** 1 Department of Orthopaedics, Sri Ramachandra Institute of Higher Education and Research, Chennai, IND; 2 Department of Orthopaedics, Orthopaedic Research Group, Coimbatore, IND; 3 Department of Biotechnology, Faculty of Engineering, Karpagam Academy of Higher Education, Coimbatore, IND; 4 Department of Orthopaedics, Government Medical College, Dindigul, IND; 5 Department of Anaesthesiology, Sooriya Hospital, Chennai, IND

**Keywords:** geriatric hip fracture, outcome analysis, fracture femoral neck, elective orthopaedic surgery, eras protocol

## Abstract

Introduction: Geriatric hip fractures are the new global pandemic. It is predicted to reach 7.3-21.3 million cases worldwide by 2050. Even with optimal care, geriatric patients suffer a higher morbidity and mortality rate when compared with the general population and often demand expensive hospital aftercare. This study aims to assess the implications of the successful adoption of the enhanced recovery after surgery (ERAS) protocol in the management of geriatric hip fractures in an Indian facility.

Methods: This is a retrospective study conducted in a tertiary care hospital in India and reported following REporting of studies Conducted using the Observational Routinely collected health Data (RECORD) guidelines. We included all geriatric patients over 60 years of age who were admitted with hip fractures for surgical management between January 2021 and January 2023. The individual perioperative components of the ERAS protocol focus on key areas such as preoperative nutritional support, effective multimodal analgesia with optimal pain control, fluid management, and early postoperative mobilization.

Results: Thirty-eight geriatric patients with a mean age of 77.5 (± 9.6) years were included for analysis. Twenty-three patients sustained intertrochanteric fractures and underwent fixation with proximal femur nailing and the remaining had 15 sustained neck or femur fractures of which 11 underwent hemiarthroplasty surgery and the remaining four underwent a total hip replacement. The mean time to surgery was 2 (± 0.2) days. Eighty-two percent (n=31) of the patients were mobilized with a walking frame within a day after surgery and were followed up after discharge with home physiotherapy. The mean time to ambulation was 2 (± 0.62) days. The mean length of stay was 4 (± 1.6) days. We had a 30-day readmission rate of 5.2% (n=2) and a 30-day mortality rate of 5.2% (n=2). The one-year mortality rate was 13% (n=5).

Conclusion: Management of geriatric hip fractures requires exceptional interdisciplinary coordination and carefully planned strategies to optimize patient care. With the implementation of the ERAS protocol, we could perceive clinical benefits in terms of early recovery and short length of hospital stay in patients with hip fractures. Further comparative studies are required, which can determine the relative importance of individual measures in the ERAS protocol and understand their longer-term outcomes in hip fracture surgeries.

## Introduction

Geriatric hip fractures could be the new global pandemic [1]. It is predicted to reach 7.3-21.3 million cases around the world by 2050 [2]. Even with optimal care, these patients suffer a higher morbidity and mortality rate when compared with the general population, and often demand expensive hospital aftercare [3]. This is mainly due to the poor prognosis owing to the increased risk of complications making hip fractures be called “the last fracture of whole life” [4]. The commonly reported risk factors contributing to the poor prognosis include non-modifiable risk factors such as advanced age, and gender along with modifiable risk factors such as smoking, alcohol consumption, diabetes, hypertension, and osteoporosis [5]. To prevent the domino effect of hip fractures on the geriatric population causing continued disability resulting in the consumption of financial, material, and human resources, emphasis on the prevention along with effective management of the fracture, once it occurs, is being highlighted [6].

Following the successful implementation and evident benefit of the enhanced recovery after surgery (ERAS) protocol in colorectal surgeries [7], it is currently being recommended and used for a variety of surgical procedures [8-13]. The promotion and implementation of the ERAS protocol have achieved satisfactory results across many surgical specialties. The initial adoption of the protocol took place on a hospital-to-hospital basis with varying success. Following national initiatives such as the Enhanced Recovery Partnership Program in the UK through collaboration with NHS Institute for Innovation and Improvement, the ERAS is now adopted across all the centers of the NHS in the UK [14]. Similarly, initiatives are being taken by many countries to effectively utilize their health services.

This study aims to assess the implications of the successful adoption of the ERAS protocol in the management of geriatric hip fractures in a tertiary care Indian facility.

## Materials and methods

We set out to explore the practicability of the application of ERAS principles in the management of geriatric hip fractures in a tertiary care hospital (Sooriya Hospital, Chennai) in India from January 2021 to January 2023. This is a retrospective study conducted and reported following REporting of studies Conducted using the Observational Routinely collected health Data (RECORD) guidelines [15]. The study was conducted after obtaining clearance from the Institutional Review Board of Sooriya Hospital.

We included all geriatric patients over 60 years of age who were admitted with hip fractures for surgical management between January 2021 and January 2023. Osteosynthesis was performed for intertrochanteric fractures using proximal femur nailing. The arthroplasty surgeries performed for fractures of the femoral neck include total hip replacement or bipolar hemiarthroplasty depending on the patient's demand, general condition, and status of the acetabulum. The surgical treatment guideline remained unchanged despite the introduction of the ERAS protocol.

The individual perioperative components of the ERAS protocol (Table 1) focus on key areas such as preoperative nutritional support, effective multimodal analgesia with optimal pain control, fluid management, and early postoperative mobilization.

**Table 1:** Components of the ERAS followed perioperatively for geriatric hip surgery patients DVT: Deep vein thrombosis; ERAS: enhanced recovery after surgery

Preoperative components	Intraoperative components	Postoperative components
Oral multimodal analgesia	Early time to surgery	Prevention of postoperative nausea and vomiting
Comorbidity optimization	Regional anesthesia	Early oral intake
Fluid optimization	Early advanced care planning	Early mobilization and breathing exercises
Preoperative nutritional support	Blood loss prevention	Scheduled alternatives to opioids
Prehabilitative chest physiotherapy		Early supported discharge
Delirium prevention		DVT prophylaxis
DVT prophylaxis		Bedsore prevention
Bedsore prevention		

Optimization of medical conditions and nutrition was started for all patients at admission. This included high-protein drinks, supplements wherever necessary along with vitamin D loading dose, and calcium for all patients. Multimodal analgesia with diligent care to avoid polypharmacy and delirium was followed. The hydration status was optimized with supervised fluid management. All patients received bedsore preventive measures (alpha bed, skin care), deep vein thrombosis (DVT) preventive measures (pneumatic compression devices, DVT stockings), and prehabilitation chest physiotherapy (respirometer, incentive spirometry) and limb mobilization exercises as appropriate along with the motivation for postoperative mobilization. Only selective specialist involvement and relevant investigations were undertaken. We conducted a family meeting to explain the condition of the patient and the necessary support system need from the family to enhance the recovery process following surgery.

All patients were taken up as the first case on the day of surgery with four hours of starvation before surgery. We preferred neuraxial anesthesia as far as possible. We used one gram of intraoperative intravenous tranexamic acid to control blood loss in most of the patients. We preferred paracetamol with regional blocks for postoperative analgesia. Early oral intake of sips of clear liquids along with in-bed mobilization and breathing exercises was started in the recovery. Patients were made to stand with walker support either on the same day or the next day and mobilized early along with twice daily postoperative physiotherapy rehabilitation. Intravenous fluids and drugs were tapered early on the second postoperative day. The above pathway was followed as a protocol and done with minimal and focused manpower and care services with minimal wastage of resources and non-medical costs.

The outcome assessed to analyze the performance of the protocol includes time to surgery, time to ambulation, length of stay, readmission rate, complications rate, and mortality rate. We used mean and standard deviation to present the continuous data and percentages for discrete data.

## Results

The case records and clinical outcomes of 38 geriatric patients with hip fractures were reviewed and included for analysis. The mean age of these patients was 77.5 (± 9.6) years with 20 male and 18 female patients. The right-side hip was commonly involved among the included patients (55.3%). Twenty-three patients sustained intertrochanteric fractures and underwent fixation with proximal femur nailing and the remaining had 15 sustained neck of femur fractures of which 11 underwent hemiarthroplasty surgery and the remaining four underwent a total hip replacement as shown in Table 2.

**Table 2:** Characteristics of patients included in the study SD: Standard deviation

Characteristics	Results (n=38)
Mean age (SD)	77.5 (±9.6) years
Sex	
Male (n)	20
Female (n)	18
Side	
Right hip (n)	21
Left hip (n)	17
Fracture type	
Neck of femur fracture (n)	15
Intertrochanteric fracture (n)	23
Procedures	
Proximal femur nailing (n)	23
Hemiarthroplasty (n)	11
Total hip replacement (n)	4

The mean time to surgery was 2 (± 0.2) days excluding 11 patients (28.9%) on double blood thinners where surgery was withheld for five days. Eighty-two percent (n=31) of the patients were mobilized with a walking frame within a day after surgery and were followed up after discharge with home physiotherapy. The mean time to ambulation was 2 (± 0.62) days. The reasons for delayed mobilization in the remaining seven patients were neurological (four patients: two patients with Parkinson’s disease, one with dementia, and one with delirium), associated fractures (two patients: one upper limb and one rib fracture), and severe chronic obstructive pulmonary disease (COPD) in one patient. The postoperative surgical drains were removed on the second postoperative day consistently. The mean length of stay was 4 (± 1.6) days as shown in Table 3.

**Table 3:** Outcome measures of the ERAS protocol for the patients included in the study COPD: Chronic obstructive pulmonary disease; SD: standard deviation; ERAS: enhanced recovery after surgery

Outcome measures	Results
Mean time to surgery (SD)	2 (±0.2) days
Mean time to ambulation (SD)	2 (±0.6) days
Mean time to drain removal	48 hours
Mean length of stay (SD)	4 (± 1.6) days
30-day readmission (n)	2
Cause of 30-day readmission	COPD exacerbation
	Urinary tract infection
30-day mortality (n)	2
Cause of 30-day mortality (age of death)	Myocardial infarction (89)
	Pneumonia (79)
1-year mortality (n)	5

We had a 30-day readmission rate of 5.2% (n=2). One patient had an acute exacerbation of severe COPD and was readmitted for its management. Other patients developed bladder outlet obstruction and urinary tract infection (UTI). Both of them were not directly related to the index surgery. Our 30-day mortality rate was 5.2% (n=2) where one patient succumbed to pneumonia at the age of 79 and another to myocardial infarction at the age of 89. The one-year mortality rate was 13% (n=5).

## Discussion

Despite the improvement in the surgical techniques in the management of hip fractures in the geriatric population, newer perioperative measures have been developed to reduce the morbidity and mortality due to the hip fracture and the surgical procedure employed in its management. The ERAS has brought a paradigm shift in perioperative care with substantial improvement in the outcome along with cost savings [16]. While the specific components of ERAS may vary based on patient characteristics and surgical procedure, the common elements of the ERAS protocol in hip surgery include preoperative education and counseling where the patients are informed about the surgery, its benefits and risks, the ERAS protocols, and the expected recovery process which helps to reduce anxiety and set realistic expectations; preoperative nutritional support to reduce the risk of postoperative complications; optimization of medications as needed including multimodal analgesic approach; prehabilitation which includes physical and functional exercises that patients can do before surgery to improve their strength and mobility to enhance recovery postsurgery; minimally invasive techniques to reduce surgical trauma; perioperative fluid management to avoid both dehydration and fluid overload; early mobilization often within a day of surgery along with physiotherapy to prevent complications such as deep vein thrombosis; early oral feeding to restore gastrointestinal function and improve patient comfort; and standardized discharge guidelines for when the patient is ready for discharge along with a follow-up plan. 

Various studies have shown that time to surgery is a critical indicator that influences the length of stay with an indirect role in increasing the risk of mortality, infection, and complications [17-19]. The mean time to surgery noted in the ERAS pathway in the meta-analysis by Liu et al. in patients with hip fracture was 28 hours [20], whereas in our study the time to surgery was 48 hours. The possible reason for the delay noted was the lack of an established treatment pathway for hip fracture cases. Al-Ani et al. in their study showed that earlier surgeries resulted in an improved ability to return to independent living among 850 patients undergoing hip fracture surgery [21]. 

The meta-analysis by Liu et al. also noted the length of stay to range from 6 to 45 days among hip fracture patients [20], while our study noted a mean length of stay of four days which is a significant reduction compared to the global standards. Similarly, the overall readmission rate noted in their study was 13% while the readmission rate noted in our study was 5.2%. Nikkel et al. in their analysis found that decreased length of hospital stay for hip fracture was associated with reduced rates of early mortality in their cohort in New York State [22]. 

The complications noted in our study were comparable to those noted in the study by Liu et al. [20]. Although a 30% mortality rate was noted following hip fracture surgery [23], our study noted a reduced mortality rate of 5.2%. Many factors could account for the increased mortality following hip fracture surgery such as time to surgery, cardiovascular disease, pulmonary disease, and malignancy [24]. In summary, both time to surgery and patient characteristics could affect the mortality following hip fracture. 

Many studies employed the length of hospital stay as the primary outcome to analyze the usefulness of the ERAS pathway [25]. Reduction in the length of stay might seem like an attractive prospect, but it is not deterministic of the patient’s recovery speed or its quality. Hence, we have utilized other outcome measures such as time to mobilization as well as the mortality rate since postoperative complications in the perioperative period might have an impact on the long-term survival of the patient [24,26]. However, the death of two patients noted in this study was unrelated to the procedure.

A hip fracture resulting in pain, associated bleeding, and immobility results in common complications such as delirium, pneumonia, UTI, venous thromboembolism, and surgical site infections [27]. The ERAS protocol has been demonstrated to reduce the risk of occurrence of these complications effectively [20,28]. Despite reducing the time to surgery and length of stay in the hospital, it has been noted that the mortality at one year in hip fracture could reach up to 30% [24]. Numerous factors contribute to mortality after hip fracture surgery. Chang et al. in their study found that time to surgery, cardiovascular disease, pulmonary disease, residential status, and malignancy were the preventable risk factors associated with mortality in hip fracture surgery [24]. 

The postoperative measures with high strength of evidence to facilitate optimal outcomes in geriatric hip fractures include supervised gradual strengthening with balance training [6]. However, other measures such as early treatment, weight-bearing exercise, home-based rehabilitation, bisphosphonate usage, and nutritional management lack sufficient evidence to have a strong recommendation in postoperative care among geriatric patients [6].

There are still some lacunae in the existing ERAS protocols such as the management of perioperative anemia, management of postoperative fatigue, and delirium [2]. Furthermore, guidelines are needed for the usage of urinary catheters in geriatric patients with lower limb fractures. To move forward with the ERAS protocol to offer the best possible perioperative care to the patients undergoing orthopedic surgery, patient-specific and procedure-specific measures must be incorporated and refined as and when new evidence emerges. 

Our study has several limitations. First, the retrospective nature of the study without a comparative cohort prevented us from comparing the exact impact of the protocol assessed to the existing standards. Second, the significance of the individual measures incorporated in the ERAS protocol could not be assessed. Finally, the small sample size of the observed cohort limits us from making any overarching conclusions about the benefit of the ERAS protocol. 

## Conclusions

Management of geriatric hip fractures requires exceptional interdisciplinary coordination and carefully planned strategies to optimize patient care. With the implementation of the ERAS protocol, we could perceive clinical benefits in terms of early recovery and short length of hospital stay in patients with hip fractures. Further comparative studies are required which can determine the relative importance of individual measures in the ERAS protocol and understand their longer-term outcomes in hip fracture surgeries.
